# Exercise reduces the risk of falls in women with polypharmacy: secondary analysis of a randomized controlled trial

**DOI:** 10.1038/s41598-025-88205-y

**Published:** 2025-02-19

**Authors:** Anna-Erika Tamminen, Risto Honkanen, Heli Koivumaa-Honkanen, Joonas Sirola, Reijo Sund, Heikki Kröger, Toni Rikkonen

**Affiliations:** 1 Kuopio Musculoskeletal Research Unit (KMRU), University of Eastern Finland, Kuopio, Finland; 2 Institute of Clinical Medicine (Psychiatry), University of Eastern Finland, Kuopio, Finland; 3 Mental Health and Wellbeing Center, Kuopio University Hospital, Kuopio, Finland; 4 Department of Orthopaedics, Traumatology and Hand Surgery, Kuopio University Hospital, Kuopio, Finland

**Keywords:** Geriatrics, Lifestyle modification, Preventive medicine

## Abstract

Polypharmacy has previously been found to increase and exercise interventions to reduce the risk of falls and fall-related injuries. In this study, women who had four or more regular medications benefitted the most from the exercise intervention and had the lowest fall risk compared to the reference group. Fall injuries among older people cause significant health problems with high societal costs. Previously, some exercise interventions have been found to reduce the number of falls and related injuries. We studied how different levels of medication use affect the outcome of an exercise intervention in terms of preventing falls. This exercise RCT involved 914 women born in 1932–1945 and randomly assigned to the intervention (*n* = 457) and control (*n* = 457) groups. Both groups participated in functional tests three times during the study. Baseline self-reported prescription drug use was trichotomized: 0–1, 2–3, and ≥ 4 drugs/day (i.e. polypharmacy group). We used Poisson regression for follow-up fall risk and Kaplan-Meier survival analysis for fractures. During follow-up, 1380 falls were reported, 739 (53.6%) resulting in an injury and pain and 63 (4.6%) in a fracture. Women with polypharmacy in the intervention group had the lowest fall risk (IRR 0.713, 95% CI 0.586–0.866, *p* = 0.001) compared to the reference group that used 0–1 medications and did not receive the intervention. Overall, the number of medications associated with the fall incidence was only seen in the intervention group. However, the number of medications was not associated with fractures in either of the groups. Weaker functional test results were associated with polypharmacy in the control group. The most prominent decrease in fall risk with exercise intervention was seen among women with polypharmacy. Targeting these women might enhance fall prevention efficacy among the aging population.

Trial Registration: The study has been registered in ClinicalTrials.gov. Trial registration number NCT02665169. Register date 27/01/2016.

**Supplementary Information:**

The online version contains supplementary material available at 10.1038/s41598-025-88205-y.

## Introduction

One-third of people over the age of 65 fall every year, half of them repeatedly^1^. In general, about a third of the falls result in injuries^2^, but these are often minor i.e. small wounds, abrasions, bruises, or other soft tissue damages^3^. In the case of older women, about 20% of the falls result in serious injuries such as joint dislocations, and head injuries with about 10% of all falls involving fractures^4^. Thus, fall-related Injuries are one of the major causes of long-term pain, disability, mortality, and even death in the elderly^5^. Falls alone increase the risk of long-term stay in a nursing home, but even more so in case of serious injuries^6^. Globally about 684,000 people die each year due to a fall^7^.

The risk of falling increases with age, with women being injured more often than men^8^. There are also many other predisposing risk factors for falls, such as a history of previous falls, poor visual acuity, slowed walking speed, impaired balance, and muscle strength^9–11^. Several diseases such as stroke and Parkinson’s disease can increase the risk of falls^9^, whereas smoking, lack of exercise, cognitive impairment, and multiple illnesses can increase that of injurious falls^10^.

In addition, polypharmacy is an independent risk factor for falls^12–14^, fall injuries^15,16^, and fractures^17,18^ in the elderly. The rate of falls is almost 20% higher for those taking more than four drugs than for those without medication^12^. Polypharmacy nearly doubles the risk of hospitalization after a fall and the risk increases according to the number of drugs in use^19^. Overall, the more drugs in use, the greater the risk of injury from a fall^15,16^.

There is no official definition of polypharmacy, but it often refers to the concomitant use of several drugs. In some definitions, it refers to the inappropriate use of drugs^20^. Polypharmacy is the most prevalent in the elderly since the use of drugs becomes more common with age. Around 30% of the population over the age of 65 use at least five drugs and about 10% use more than ten drugs daily^21^. Female gender, chronic diseases, and multiple doctor visits are associated with polypharmacy^22^. Thus far, the effect of polypharmacy on the outcome of an exercise intervention for fall prevention in the elderly has not been previously studied.

Exercise interventions have been shown to reduce the number of falls^23,24^, as well as fall-related injuries and fractures in the elderly^25,26^. In general, a multi-component intervention appears to be more effective than a single-component intervention in fall prevention^23^. According to a systematic review and meta-analysis, the effect of an intervention could be enhanced by increasing the amount of exercise to more than three hours per week and incorporating balance training into it^24^. For example, a series of gentle physical exercises and stretches i.e., Tai chi can improve balance and reduce the number of falls in the elderly^27,28^.

The study aimed to investigate how different levels of medication use are associated with the efficacy of fall prevention exercise intervention. In addition, we wanted to find out how polypharmacy and exercise intervention are associated with functional capacity and physical fitness measured by functional tests.

## Materials and methods

### Study design and participants

The Kuopio Fall Prevention Study (KFPS) is a two-year randomized controlled trial conducted as a part of the Kuopio Osteoporosis Risk Factors and Prevention (OSTPRE) study. It is carried out at the University of Eastern Finland (UEF). The study protocol and the main results have been previously published in detail^29,30^.

The KFPS cohort consists of women born between 1932 and 1945, living within 10 km of the city center of Kuopio, willing and eligible for the 2-year exercise intervention. An information letter about the study was sent to 4262 women in 2016–2017. Preliminary screening excluded women who did not meet the criteria i.e., women with unstable angina pectoris, severe lung disease, moderate or severe dementia.

In total 914 women were randomized. Out of these, 582 women belonged in the ongoing OSTPRE follow-up cohort born between 1932 and 1941. Additionally, 332 women born between 1942 and 1945 were recruited from outside the OSTPRE cohort. The final allocation to intervention (*n* = 457) and control (*n* = 457) groups were performed after baseline health examination. Block randomization was used to guarantee equal allocation to both groups.

### Exercise intervention and health education

During the first year, both the intervention and control groups received health education in two sessions, which included written and oral information on fall prevention, nutrition, and home safety. In addition, information was provided on free exercise opportunities for the public in the Kuopio city region.

The intervention group underwent a Tai Chi course led by an expert and supervised gym training for the first six months, both of which took place once a week. The gym workout was conducted as an intensive circuit training led by two physiotherapists focusing on strengthening large postural muscle groups. The Tai Chi course focused on strengthening the muscles of the lower limbs, as well as exercising balance, coordination, and range of motion. In the elderly, this kind of multi-component intervention has proven efficient in preventing falls, injuries, and fractures^23,24,26^.

After the initial six-month supervised workout period, the intervention group continued the gym routine without guidance, but with free-of-charge access to municipal training facilities offered by the city of Kuopio. During the last year of the study, the intervention group had the opportunity to exercise at their own expense at a low cost. Meanwhile, the control group was free to continue their usual exercise activities for two years. During the follow-up, no serious adverse events were reported related to training sessions. For detailed events description, see the RCT main results^30^.

### Clinical measurements and functional tests

Both study groups visited the Kuopio Musculoskeletal Research Unit three times during the study i.e., at the beginning and after 12 months and 24 months. Clinical measurements including weight, height, waist circumference, and functional tests were performed. These functional tests included an isometric leg extension test, a dominant hand grip strength test, and a timed-up-and-go test. Maximal isometric leg extension was performed in a sitting position, three times for each leg with a 65% flexion. Hand grip was measured three times from the dominant hand with a 30-second rest in between. The timed-up-and-go test consists of a patient getting up from a chair, walking three meters, turning, returning, and sitting on the chair again.

### Polypharmacy, falls, and fall-related injuries

In the present study, polypharmacy refers to the regular use of four or more prescribed drugs. The subjects reported their prescribed medication use in the baseline questionnaire. In the questionnaire, the women chose the drug groups they used from the given options (Appendix 1). Individual drugs were not asked.

Falls were monitored through diaries and biweekly SMS (short message service) text messages. Mobile phone text messages were sent automatically every other week with the question “Have you fallen during the last two weeks?”. The answer options were yes/no. When the answer was positive, the person was interviewed by phone about this fall event. The falls were recorded according to the WHO International Classification of Diseases (ICD-10). All injuries related to a fall, with slipping, tripping, or falling from a height of less than one meter, resulting in self-reported at least moderate pain and tenderness, were regarded as injuries caused by a fall.

In addition, subjects recorded falls in a study diary. The date, place, time, and cause of all falls, as well as the place of treatment and hospitalization for the falls requiring medical treatment, were recorded. The diary was returned every three months. National medical records (from the Care Register for Health Care and Register of Primary Care Visits) were checked for fractures and medical history since the number of self-reported injuries has previously been found to be inaccurate^31^.

### Statistical analysis

According to baseline self-reported prescription drug use, women were divided into six groups for the statistical analyses: intervention and control groups and further subgroups 0–1 drug users, 2–3 drug users, or 4 or more drug users (i.e. the polypharmacy group). Due to the age of this cohort, there is a very marginal number of women without medication (*n* = 77, 8%), therefore none-users and 1 medication users have been combined into one group. Differences between these groups in functional tests and baseline characteristics were examined by analysis of variance (ANOVA) and chi-square test. The results of the functional tests were compared using analysis of variance in each time point. Person years were calculated by summing up the total number of years that each woman participating in the study has been under observation.

The fall frequency between study groups was compared using the Poisson regression model. The reference group was set accordingly to women who used 0–1 drugs and did not receive the intervention. A total of 15 women did not respond to any of the fall questionnaires and were therefore excluded from the analysis. The cumulative probability of the first fractures during follow-up time was studied with Kaplan-Meier survival analysis. In all analyses, *p* < 0.05 is considered statistically significant.

## Results

The mean (SD) follow-up of the study subject (*N* = 914) was 1.9 years, the median being 2 years. At baseline, altogether 8% of women (*n* = 77) had no regular medication. The mean number of medications for the other women was 3.0. Out of the latter, 25%, *n* = 226 used 0–1 drugs, 39%, *n* = 358 used 2–3 drugs, and 36%, *n* = 330 belonged to the polypharmacy group (≥ 4 or more drugs). Only higher weight and BMI from the studied baseline characteristics were significantly associated with the number of drugs (Table 1).

**Table 1:** Baseline characteristics for 914 women with their respective mean values.

	0–1 drug	SD	2–3 drugs	SD	4 or more drugs	SD	*p* (ANOVA*)
n	226		358		330		
Age (years)	76.4	3.3	76.3	3.2	76.9	3.2	0.063
Weight (kg)	66.0	10.9	68.1	11.3	72.2	13.7	< 0.001
Height (cm)	158.9	5.8	159.1	5.6	158.6	7.8	0.663
BMI (kg/m^2^)	26.1	5.8	26.9	5.6	28.5	7.8	< 0.001
Smoker**	8 (3.5%)		5 (1.4%)		4 (1.2%)		0.098

*Analysis of variance.

**Chi-squared test.

Subjects reported considerably fewer chronic health disorders than prescribed medications. For example, 661 women report the use of cardiovascular drugs while only 81 women report having a circulatory system disease diagnosed within two years of baseline. This was seen in all drug groups and chronic health disorders. The self-reported diseases and drugs at baseline are presented in more detail in the supplement (Supplement Tables 1 and 2).

Functional tests were performed at baseline, at 12 months, and 24 months. Weaker test results were associated with polypharmacy. At baseline, the results of the intervention and the control groups were similar. The polypharmacy group who did not receive intervention had the worst results in the functional test regardless of the test and the time point (Table 2).

During the first year, grip strength and leg extension results improved in all drug groups, and grip strength results ceased to be significantly different between these groups. Only the timed up-and-go test deteriorated: 3% for 0–1 drug users, 1% for 2–3 drug users, and 4% for polypharmacy users (Table 2). Further, almost all the functional tests improved more in the intervention group than in the control group.

In the second year, the results of functional tests had deteriorated in all drug groups from the 12-month results. The TUG test results deteriorated the most: 8% in the 0–1 drug group, 11% in the 2–3 drug group, and 11% in the polypharmacy group. The test results were similar to those at baseline, being best in the 0–1 drug users and the weakest in the polypharmacy group. Only in the intervention group of 0–1 drug users, the results were still better than at baseline except for the TUG test. The 2–3 drug users of the intervention group performed worse than the controls and worse than at the baseline.

The drop-out rates for the first 12 months were 4%, *n* = 9 for 0–1 drug users, 7.3%, *n* = 26 for 2–3 drug users, and 8.8%, *n* = 29 for the polypharmacy groups, and for the 2-year follow-up, 5.3%, *n* = 12, 8.4%, *n* = 30, and 10.3%, *n* = 34, respectively. The drop-out rate in the first year was higher than in the second year in all groups.

**Table 2:** Functional capacity according to test results means at baseline, 12 months, and 24 months in those using 0–1, 2–3, and 4 or more drugs for both study groups and the statistical difference between these groups at a given time point.

		0–1 drug			2–3 drugs			4 or more drugs			*p* (ANOVA)
		Mean	Intervention		Mean	Intervention		Mean	Intervention		
			Yes	No		Yes	No		Yes	No	
Baseline measurements											
N		226	116	110	358	164	194	330	178	152	
Grip strength (kg)		26.1	26.1	26.2	26.6	26.6	26.6	25.4	25.3	25.6	0.01
Isometric leg extension strength (kg)	Left	30.2	30.1	30.3	30.1	29.9	30.3	28.4	29.1	27.7	0.005
	Right	30.6	30.4	30.8	29.9	29.8	30.0	28.7	29.4	28.1	0.016
TUG (s)		9.4	9.5	9.3	9.6	9.6	9.6	10.1	10.1	10.2	0.191
12-month measurements											
N		217	113	104	332	156	176	301	161	140	
Grip strength (kg)		27.1	27.7	26.5	27.0	26.8	27.1	25.9	26.1	25.6	0.146
Isometric leg extension strength (kg)	Left	31.9	32.0	31.7	30.8	31.1	30.4	29.4	30.2	28.4	0.001
	Right	31.9	32.1	31.6	30.6	30.6	30.6	29.8	31.0	28.3	0.009
TUG (s)		9.7	10.0	9.3	9.7	9.7	9.8	10.5	10.4	10.5	< 0.001
24-month measurements											
N		214	110	104	328	154	174	296	158	138	
Grip strength (kg)		26.1	26.4	25.9	26.2	26.0	26.3	25.1	25.1	25.0	0.015
Isometric leg extension strength (kg)	Left	29.6	30.1	29.2	28.5	28.5	28.5	27.3	28.1	26.4	0.002
	Right	30.9	31.5	30.3	29.9	29.8	30.0	29.1	30.0	27.9	0.015
TUG (s)		10.5	10.7	10.2	10.8	11.0	10.6	11.7	11.6	11.8	< 0.001

Study subjects reported 1380 falls in the two-year follow-up and 46% of these resulted in injury. Altogether 61%, *n* = 558 of women fell at least once, and 34.5%, *n* = 315 fell several times. Table 3 shows the number of falls and fall risks with the reference group of 0–1 drug users from the control group. The lowest 2-year fall risk was observed in women with polypharmacy who participated in an exercise intervention (IRR 0.713, 95% CI 0.586–0.866, *p* = 0.001). The second lowest fall risk was in the intervention group that used 2–3 drugs (IRR 0.811, 95%CI 0.671–0.981, *P* = 0.031). In the control group, the number of drugs did not affect the fall risk. Also, the use of 0–1 medication did not affect the fall risk in the intervention group.

**Table 3:** Fall risk over a two-year follow-up period compared to a reference group using 0–1 drug with no intervention. Adjusted for age and follow-up time.

	*N*	Mean follow-up time (years)	Number of falls	Falls/1000 person-years	IRR	95% CI	Sig.
4 or more drugs Intervention	175	1.86	213	647/1000	0.713	0.586–0.866	0.001
4 or more drugs No intervention	151	1.86	256	906/1000	1.022	0.847–1.233	0.824
2–3 drugs Intervention	162	1.93	238	752/1000	0.811	0.671–0.981	0.031
2–3 drugs No intervention	188	1.87	291	802/1000	0.879	0.732–1.054	0.165
0–1 drug Intervention	114	1.87	190	876/1000	0.968	0.792–1.183	0.751
0–1 drug No intervention	109	1.89	192	924/1000	1	ref.	

During the follow-up, 63 women suffered a fracture due to a fall. Table 4 shows the number of women with fractures during follow-up time. The control group had the highest percentage of women with fractures, the highest percentage belonging to the 0–1 drug users (11.8%, *n* = 13) followed by the polypharmacy group (9.3% *n* = 14) and the 2–3 drug users (6.2%, *n* = 12). The intervention group had slightly fewer women with fall-related fractures than the control group, the lowest percentage belonging to the 0–1 drug users (4.3%, *n* = 5), and the highest in the 2–3 drug users (6.1%, *n* = 10) and the polypharmacy group being in the middle (5.1%, *n* = 9). Polypharmacy did not significantly affect the number of fractures (p (Kaplan-Meier survival analysis) = 0.717).

**Table 4:** The number of women with fractures during follow-up time.

	*n*	Number of women with fractures	Follow-up time in days
4 or more drugs Intervention	177	9 (5.1%)	711
4 or more drugs No intervention	151	14 (9.3%)	699
2–3 drugs Intervention	164	10 (6.1%)	706
2–3 drugs No intervention	194	12 (6.2%)	705
0–1 drug Intervention	116	5 (4.3%)	716
0–1 drug No intervention	110	13 (11.8%)	678

## Discussion

In this randomized controlled trial on women between 76 and 89 years of age, exercise intervention reduced falls. This fall reduction effect due to exercise was greater (− 29%) in women with polypharmacy (4+ drugs per day) (*p* = 0.001) than in women with fewer medications. Whereas in the control group with polypharmacy but without exercise, the number of medications did not affect (+ 2%) fall risk.

In 2019, according to the Social Insurance Institution of Finland, cardiovascular drugs were prescribed the most in Finland, followed by anti-inflammatory drugs^32^. These statistics, however, did not consider age or gender. In the present study, cardiovascular drugs were similarly the most used (by 36% of the study subjects), followed by the drugs for endocrine and reproductive systems (22%). It may be that their higher use of endocrine than anti-inflammatory drugs is due to our study subject being aging women, but also diabetes and the use of diabetic drugs become more common at higher ages^33^. In addition, all the subjects were postmenopausal and most of them belonged to the OSTPRE study where women reported higher hormone replacement therapy use (27%) than usual in osteoporosis in Western countries. Nevertheless, a previous study found that the number of self-reported estrogen use was consistent with prescription data from the Social Insurance Institution of Finland^34^. Women in KFPS were shown to have better physical and mental well-being, functional capability, and higher sociodemographic status than the non-participants^35^. In the present study, they also reported significantly fewer diagnosed conditions than prescribed medications. This may be due to their good physical and mental well-being, indicating only a sporadic need for medication or the good treatment outcome of their chronic health disorders and/or recall bias. Nevertheless, this needs further exploration.

The difference in leg extension force, grip strength, and timed up-and-go test results were statically significant throughout the follow-up between the three drug groups at almost all given time points. The results of all groups improved during the follow-up period, but the best results were mainly in the 0–1 drug users and the weakest was consistently the polypharmacy group. Other studies have found similar results. Among those using more than five drugs, time up-and-go test results have been reported to be slower^36^ and the grip strength to be weaker^37^ compared with the other drug groups.

Previous studies have shown that the good outcomes of an exercise intervention can be expected to decline after the intervention; Henderson et al. found that the results of the Short Physical Performance Battery weakened, and the walking speed slowed near to the baseline level about a year after the exercise intervention in older adults^38^. A similar result was found in the present study. Only 0–1 drug users in the intervention group had two 24-month tests slightly better (grip strength and right leg extension) than at baseline. The intervention group had improved results in the 12-month tests. Thereafter, their free use of the city’s recreational sports facilities expired resulting in their decreasing use of these exercise premises and decline in the 24-month tests. Previous physical activity and good functional ability were related to continuing to use municipal training facilities after the end of guided exercise^39^.

Study subjects were asked about falls biweekly by SMS text messages, which enabled us to verify recent falls. In previous studies, the number of falls or injuries caused by falls has been determined retrospectively for example through interviews^40^ or hospital registry^16^. The number of self-reported hip fractures has previously been found to be inaccurate^31^. Probably due to this, in the present study, the overall number of falls reached a higher count than anticipated. A fall occurring within two years was reported by 61% of our study population, compared with another prospective cohort study with only 16.5% of the subjects reporting a fall within a year^40^.

It has previously been found that polypharmacy increases the risk of falls and fall-related injuries^12–16^. The number of drugs has shown a dose-dependent relationship with falls^15,16^. However, in the present study, the number of daily drugs had no association with fall incidence among women in the control group. Instead, exercise intervention had the highest fall prevention efficacy among women with polypharmacy. However, among the 0–1 drug users, the exercise intervention did not seem to affect falls. All this emphasizes the importance of identifying and targeting fall prevention for the most potential risk characteristics at hand.

The findings, that the fall risk in the elderly who participated in an exercise intervention decreases have also been reported by previous studies^41^. Polypharmacy has been associated with poor physical fitness and an increased risk of falling^42,43^. Thus, the physical condition among those using a maximum of one daily drug can be so good that their fall risk remains lower regardless of their intervention status.

The strength of this study was a sample size of 914 subjects, which can be considered large for any exercise intervention RCT. In addition, the study protocol included comprehensive phone interviews with the biweekly SMS tracking of falls and related injuries, to get their number as close as possible to the true occurrence of falls. However, this RCT had classical selection bias towards women with better physical and mental well-being with more interest concerning their health, which has been reported previously^35^. As the physically inactive women were less likely to participate, this can result in better initial functional tests and can limit the generalizability of our findings. As multimorbidity has been associated with low levels of physical activity^44^ the participants of the present study might have somewhat healthier—with less polypharmacy—than in this age in general. In addition, the baseline questionnaire may have affected the number of reported drugs. Women chose the drugs they used from the given drug groups. As a result, drugs may have been left unreported if a suitable group was not available.

In conclusion, a prominent decrease in fall risk with exercise intervention was seen among women with polypharmacy. Targeting these women might enhance fall prevention efficacy among the aging population.

## Electronic supplementary material

Below is the link to the electronic supplementary material.

Supplementary Material 1

## Data Availability

The data underlying this article will be shared on reasonable request to the corresponding author.

## Declarations

*Consent*.
