# Supplementary material for: Exercise reduces the risk of falls in women with polypharmacy: secondary analysis of a randomized controlled trial
Source: Sci Rep. 2025 Feb 19;15:6009. doi: 10.1038/s41598-025-88205-y (PMC11839976; doi:10.1038/s41598-025-88205-y)
Supplement: Supplementary file 1 — Supplementary Material 1 [file 41598_2025_88205_MOESM1_ESM.pdf]

Exercise reduces the risk of falls in women with polypharmacy: secondary analysis of a randomized controlled trial

Tamminen A, Honkanen R, Honkanen-Koivumaa H, Sirola J, Sund R, Kröger H, Rikkonen T.

Appendix 1

Use of medications

Are you currently using medications prescribed by a doctor? (Circle all options that apply to you.)

0 I do not have regular medication.

1 yes, I regularly use the following:

1 heart medicine

2 blood pressure medication

3 other circulatory medicine

4 anticoagulant

5 a diuretic

6 cholesterol medication

7 dementia medicine

8 antiepileptic drug

9 antidepressant

10 remedies for anxiety

11 sleeping pills

12 antipsychotics

13 insulin

14 other diabetes medications

15 thyroid medication

16 pain medication

17 cortisone tablets

18 cortisone spray

19 asthma medicine

20 osteoporosis medicine

21 cancer medicine

22 rheumatism medicine

23 hormone replacement therapy

24 other, which

Table 1. Number of women reporting diseases diagnosed within two years from baseline and statistical differences between groups. Classification of Diseases adapted from the International Statistical Classification of Diseases and Related Health Problems (ICD)

	0–1 drug	2–3 drug	4 or more drugs	p (Chi-squared test)
Neoplasms	6	12	10	0,941
Endocrine, nutritional, and metabolic diseases	6	20	19	0,383
Diseases of the nervous system	2	7	5	0,591
Diseases of the eye, adnexa, and of the ear, and mastoid process	3	11	12	0,288
Diseases of the circulatory system	7	28	46	0,01
Diseases of the digestive system	3	5	7	0,724
Diseases of the musculoskeletal system and connective tissue	7	25	32	0,061
Others	7	13	18	0,547

Table 2. Number of women using prescription drugs at baseline. Classification adapted from ATC codes.

	0-1 drug	2-3 drug	4 or more drugs	Total (percentage of all women)
Anti-inflammatory drugs	11	83	167	261 (29%)

Central nervous system drugs	6	53	118	177 (19%)
Cardiovascular drugs	75	274	312	661 (72%)
Endocrine and reproductive system drugs	31	158	211	400 (44%)
Respiratory drugs	8	48	102	158 (17%)
Gastrointestinal drugs	3	14	20	37 (4%)
Others	9	38	36	83 (9%)
